# Reframing food regulation within nutritional ecology: biocultural evidence and indigenous food systems in Australia and Aotearoa

**DOI:** 10.3389/fnut.2026.1900712

**Published:** 2026-07-17

**Authors:** Luke B. Williams

**Affiliations:** 1 School of Agriculture and Food Sustainability, University of Queensland, Brisbane, QLD, Australia; 2 School of Chemistry and Molecular Biosciences, Faculty of Science, University of Queensland, Brisbane, QLD, Australia

**Keywords:** decolonising food systems, food safety, history of safe use, indigenous food sovereignty, indigenous knowledge systems, native foods, traditional ecological knowledge, traditionally used foods

## Abstract

Food safety regulation governing traditionally used foods is grounded in Western epistemological concepts that do not accommodate Indigenous knowledge systems. In Australia and Aotearoa, determining whether a traditionally used food can enter commercial markets requires documented evidence of a history of safe use, a standard that treats knowledge as legitimate only once it has been transcribed, rendering tens of thousands of years of Indigenous oral knowledge inadmissible. Despite growing commercial interest in native foods with a history of traditional use, Indigenous peoples remain significantly underrepresented in an industry built on their knowledge. In this Perspective I argue that this standard is not the neutral evidentiary threshold it is designed to be, and that it is also scientifically incomplete when viewed through the lens of nutritional ecology, which holds that diet and safety are inseparable from their ecological and cultural context. Excluding orally held knowledge from safety assessment means losing precisely the factors that determine how a food functions in practice, including preparation, combination, seasonality, and the accumulated record of sustained human exposure. Drawing on Aboriginal food systems and Indigenous epistemological principles, I propose a biocultural evidence approach built on three principles: a dedicated regulatory category for traditionally used foods, Indigenous authority over knowledge validation, and evidence requirements proportionate to actual risk. Recognising Indigenous food knowledge in this way would advance food sovereignty by allowing communities to participate in markets on their own terms, supporting cultural continuity and the community wellbeing outcomes that flow from self-determined participation.

## 1 Introduction

The legal and regulatory systems imposed on Indigenous peoples through colonisation rarely accommodate Indigenous ways of knowing and being, and food safety regulation is no exception. Under Western food regulatory systems, such as those governing Australia and Aotearoa (New Zealand), the frameworks that are used to determine whether a traditionally used food can enter commercial markets privilege documented evidence of a history of safe use, a standard that assumes knowledge is only legitimate when it has been transcribed (1). This standard is not a neutral evidentiary threshold, especially for Indigenous communities who have developed sophisticated knowledge systems that encode food safety, preparation practices, and ecological understanding through oral tradition, language, and various cultural expressions. In practice, this standard functions as a colonial structure, rendering tens of thousands of years of lived knowledge inadmissible while accepting documented evidence from historical records and observers’ journals as valid evidence (1).

Nutritional ecology recognises diet and health as inseparable from ecological and cultural context (2), yet current food safety regulatory frameworks that are rooted in Western scientific epistemologies routinely strip foods from precisely these contexts (1), requiring decontextualised documentation that Indigenous knowledge systems, by their very nature, cannot provide. These frameworks therefore work to isolate traditional food use from its relational, historical, and ecological foundations, actively creating a barrier that excludes Indigenous peoples from participating in economies developed from their traditional food practice.

Within Australia, native foods that have a long history of use in Aboriginal communities, such as lemon myrtle, wattleseed, and Kakadu plum, are receiving growing commercial attention from primary producers and health food manufacturers exploring their nutritional and functional qualities (3). Beyond their commercial potential, these food systems are ecologically sophisticated, drought tolerant, and biodiversity rich, representing a largely untapped resource for sustainable food system development in an evolving climate (4). This presents an opportunity for Indigenous economic self-determination and environmental stewardship, yet the industry and control of these systems remains overwhelmingly non-Indigenous, with reports indicating that Indigenous-owned businesses generate only 1% of the value of an industry built on Indigenous knowledges (5).

This exclusion is not simply a market access problem. The regulatory barrier undermines the ability of Indigenous peoples to maintain control over their own food systems, eroding food sovereignty rights and limiting the cultural revitalisation that flourishes when communities participate in markets on their own terms (6), a form of economic participation that the United Nations Declaration on the Rights of Indigenous Peoples recognises as a self-determination right (7). It also devalues traditional knowledges at a time when the Australian government has committed to elevating Indigenous knowledge systems across society (8).

Grounded in nutritional ecology, which recognises that food use is far more than consumption (2), this Perspective argues that long-term human use within specific ecological and cultural contexts constitutes a form of biocultural evidence (9) that current food safety frameworks fail to operationalise. It proposes a biocultural evidence approach that offers a culturally appropriate and epistemologically coherent basis for safety assessment, and examines how the requirement for a documented history of use embeds a Eurocentric assumption that only transcribed knowledge counts as evidence, and how this functions as a colonial structure that prevents Indigenous peoples from exercising their right to economic self-determination.

Within Australia and Aotearoa specifically, no Indigenous peoples are currently involved in Food Standards Australia New Zealand’s (FSANZ) assessment of traditional use claims or represented on the FSANZ Board, a gap the agency has acknowledged in its own impact analysis (10). The approach proposed here offers an alternative structure that centres Aboriginal, Torres Strait Islander, and Māori peoples as the authority over what constitutes legitimate traditional knowledge and as the primary voice in determining whether a food should be recognised as traditional and therefore subject to a culturally appropriate assessment pathway, rather than left to seller self-assessment or relegated to the novel food framework, one that values Indigenous knowledge as a legitimate evidentiary source and supports the structural inclusion of Indigenous peoples in an industry built on their knowledges.

As an Aboriginal researcher working at the intersection of food systems, food policy, and food toxicology, I ground this paper in an Aboriginal perspective operating within Australian policy. However, the regulatory tensions described throughout are not unique to Aboriginal Australia. The Torres Strait Islanders in Australia and the Māori in Aotearoa, who are similarly governed by FSANZ and share the same documentary evidentiary standard, face equivalent barriers to developing their traditionally used foods for commercial markets (11). The structural pattern of Western regulatory frameworks privileging transcribed knowledge over oral tradition extends to other colonised and post-colonial contexts, but that broader comparative work is beyond the scope of this paper.

## 2 Traditional food systems and the limits of decontextualised safety assessment

Food safety assessment of traditionally used foods typically concentrates on quantitative measures of composition, such as the identity and levels of known compounds of concern, nutritional value, and the potential for adverse effects. This assessment is often performed on an isolated plant component, such as the edible flesh of a ripe fruit, while completely overlooking other plant components or stages of growth. While this information is necessary, it is insufficient on its own to understand how a food functions in a diet (12). As an example, the bush tomato (*Solanum centrale*) contains solanine throughout the plant, and the unripe fruit is toxic while the ripe fruit is safely consumed and prized for its flavour. Indigenous knowledge of this plant correctly identifies that the unripe fruit and other plant components should be avoided as a source of food, while making use of the otherwise toxic components in medicinal practices (13), an understanding that would be entirely overlooked and not reported if a dietary assessment were limited to a ripe fruit sample purchased from a commercial supplier.

Fully contextualising food safety requires understanding how a food is selected, prepared, and consumed, whether it is consumed in combination with other foods, and whether any known contraindications exist, as is often the case where plants, especially those that have a history of traditional use, serve dual roles as food and medicine (14). This is precisely where Indigenous food knowledge should be recognised in the risk assessment of traditionally used foods. Aboriginal food systems offer a primary example of this relational understanding, where 65,000 years of continuous occupation across some of the harshest environments (15) has been sustained by sophisticated knowledge systems governing harvest, preparation, seasonal availability, ecosystem relationships, and safe consumption of the foods that are currently being developed for commercial markets.

Aboriginal food knowledge systems constitute sophisticated bodies of knowledge that have been accumulated across generations and transmitted orally through language, story, and practice to encode safety information, ecological understanding, and nutritional knowledge in ways that are inseparable from the foods themselves (9, 16). Colonial disruption has been especially acute in Australia, where Aboriginal people were removed from Country, children were taken from families, language and culture were suppressed, and rationing systems controlled what Aboriginal people had available to eat (17). This disruption may have altered traditional food practices, but it has not erased the evidence of them. Our food ways persist in our stories, in art, and in living knowledge that continues to be passed between generations.

This is where I argue that contemporary regulatory frameworks continue colonial patterns of epistemic imperialism. Under the current system, explorers’ journals and historical cookbooks are accepted as evidence of a history of safe use, while the knowledge held by those who have used these foods for tens of thousands of years is not (1). Most significantly, the imposed standard sits in direct tension with risk assessment principles that treat sustained human exposure without adverse effect as meaningful evidence of safety (12, 18), principles the current framework claims to uphold but fails to operationalise when that exposure history exists in oral rather than written form.

Within Australia and Aotearoa, traditionally used foods do not have a defined assessment standard. Instead, a food supplier wishing to sell a traditional food item to commercial markets can self-determine whether they believe their food item is traditional or not. In this situation, sellers are responsible for deciding whether a food has a history of human consumption and bear full liability for that determination should an adverse outcome occur (19). FSANZ has itself acknowledged its structural inability to recognise Indigenous culture and food expertise. A draft regulatory impact statement, Modernising the FSANZ Act, stated explicitly that “FSANZ’s objectives do not include a recognition of indigenous culture and food expertise” (20). The finalised version softened this, redescribing the problem as an absence rather than a structural gap, but also acknowledged that “there are no operational arrangements in place that would support this ongoing dialogue, such as formal representation of Indigenous culture and expertise on the FSANZ Board” (10). This is an admission that FSANZ lacks not only the legislative objective but any operational mechanism to begin addressing it.

The trajectory from draft to final document illustrates a pattern that extends further. The subsequently released FSANZ *2030 Roadmap* contains no mention of traditionally used foods, culture, Indigenous expertise, or traditional knowledge systems, essentially omitting the issue entirely from its strategic priorities through to 2030 (21). This is particularly striking given that the Australian Federal Government has now named “Elevating Aboriginal and Torres Strait Islander Knowledge Systems” as one of five National Science and Research Priorities (8), making FSANZ’s silence a direct contradiction of a whole-of-government commitment.

Recognising alternative forms of evidence is not without precedent. Peru recognises traditional preparation practices and regional variability in its legal framework for traditionally used foods (10), and Health Canada accepts evidence presented through oral traditions (22). Within Australia, the Therapeutic Goods Administration, which operates under a more stringent evidentiary standard than food regulation, has accepted oral evidence of traditional medicine use since 2022 (23). The two regulators differ in statute, harm profile, and evidentiary threshold, but these differences do not defeat the analogy: if a higher-risk regulator has found a workable pathway for oral evidence, food regulation’s refusal to do so reflects institutional priority and legislative will rather than practical necessity.

The consequence is a denial of economic self-determination. The current framework prevents Aboriginal peoples from participating in an industry built on their knowledge, on terms they had no say in setting and cannot meet, while enabling those with capital and legal infrastructure to profit from knowledge systems they cannot legitimately claim. The regulatory barrier is one structural contributor among several, compounding historical disadvantages in capital access, land tenure, and supply chain infrastructure (24), but it is the contributor that government has the most direct power to address.

By eroding self-determined participation in food markets, the current framework directly undermines Indigenous food sovereignty and the cultural, economic, and community wellbeing outcomes that flow from it. The biocultural evidence approach proposed in the following section responds to this failure, offering a pathway grounded in Indigenous epistemologies and leadership that establishes history of use on appropriate terms without compromising the integrity of safety assessment.

## 3 Biocultural evidence principles for regulatory reform

Biocultural evidence, as the organising concept for a reformed assessment pathway, examines the interaction between human biology, culture, and ecology. It allows us to understand a “history of use” in greater context by examining how a food is used, how it is understood, and importantly, how it affects the broader systems that it is a part of. Regarding traditionally used foods, a biocultural lens allows us to understand the scope of use for a particular food, including whether there are preparation techniques, detoxification steps, and whether there are known adverse effects that accompany the use of the plant. This includes an understanding of the ecological impact, such as how a particular plant grown in one region differs from another, how the combination of plant varieties may impact bioavailability or safety, or how ecological management practices such as burning affect the growth and ultimately the composition of plant foods (25). The cycad nut (*Cycas armstrongii*), a staple food for various Aboriginal peoples across northwest Australia, Cape York, and Arnhem Land, illustrates both dimensions. Controlled burning increases fruit yield and ensures continuous supply, while the unprocessed nut contains high levels of cycasin, requiring careful preparation, including soaking in running water to leach out these toxins, before the nut can be safely consumed as a flour (26).

For Aboriginal peoples in Australia, this history of use has been developed over tens of thousands of years through ecological stewardship of the plants and the environment (9, 16). This is not anecdotal or informal knowledge; it is a structured evidence base that has been implemented and practiced over a long period of time (27). These practices are often not performed in isolation, with similar practices being found among various Aboriginal communities and language groups across the continent. Genomic research confirms that Aboriginal Australians represent a highly genetically diverse population, with substantial variation between communities and regions (28). Although knowledge systems will vary across regions, use and tolerance of these foods can be demonstrated across a genetically diverse population, an aspect that directly satisfies food safety risk assessment principles around sustained and varied human exposure (18).

The safety of traditionally used foods should be assessed under their own regulatory category, rather than leaving public health decisions to sellers who may lack the technical capacity to assess safety accurately or who face commercial pressures that discourage adequate safety assessment. Three principles form the conceptual model underpinning the proposed approach: a dedicated regulatory category for traditionally used foods, Indigenous authority over knowledge validation, and proportionate evidence requirements. For a dedicated assessment pathway for traditionally used foods to be successful, it needs to be developed in consultation with Indigenous people. At minimum, it should operate with Indigenous advisors as a reference group, a concept that has been implemented by other government departments seeking to embed Indigenous perspectives in their decision-making processes (29–32).

It is important to understand how the current regulatory framework operates differently for different market actors. Well-capitalised businesses can commission formal safety assessments, securing documented evidence of due diligence that both protects them legally should an adverse outcome occur and provides consumers and regulators with greater confidence in the safety of their products. Small Indigenous enterprises typically lack both the food safety capacity to determine what assessment is required and the financial resources to commission it (24). They enter the market through the self-assessment pathway, which they are legally entitled to do (19), but without the protective cover of documented due diligence. The framework appears to offer equal access but produces structurally unequal outcomes. Under the present self-assessment pathway, small Indigenous enterprises bear full liability for public health outcomes they are not equipped to assess, while well-capitalised businesses absorb that risk through legal and financial infrastructure. A dedicated assessment pathway for traditionally used foods, grounded in biocultural evidence principles, would improve this situation for all parties: consumers would have greater certainty about the safety of foods they purchase, Indigenous businesses would have a clear and culturally appropriate pathway for demonstrating due diligence, and FSANZ would be fulfilling its core public health mandate.

**Figure 1:**
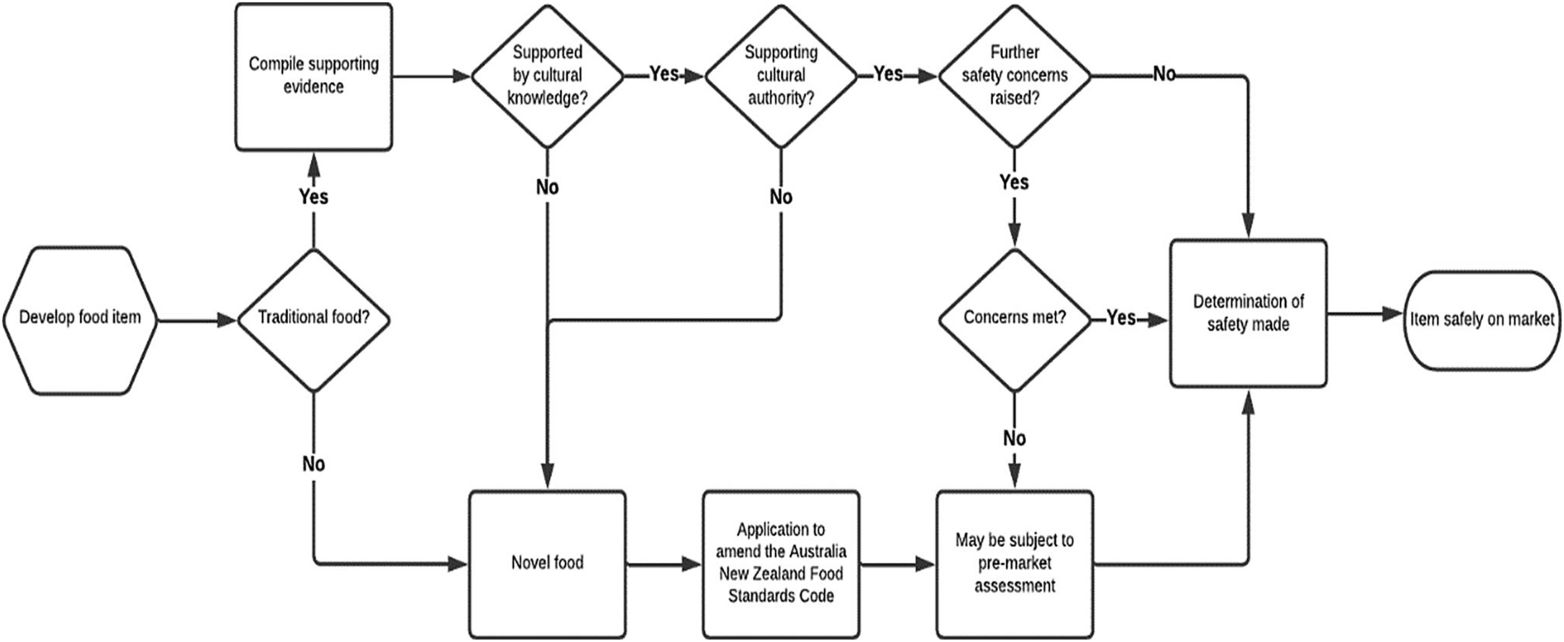
Proposed assessment pathway for traditionally used foods incorporating biocultural evidence and cultural authority principles. Full detail for each step has been described elsewhere (1). Full detail for each step has been described elsewhere. Reproduced from (1), licensed under CC BY 4.0.

As is discussed in more detail below, safety screening remains a needed process for foods entering commercial markets. However, Indigenous people should be positioned to make the decision as to whether a food is traditional to their culture or not – this does not require a trained toxicologist or government regulatory bureaucrat. It requires culturally competent individuals to determine whether a food has traditional use within a specific cultural context. The evidence that is provided to indicate traditional use should also be scrutinised by Indigenous knowledge holders, not bureaucrats operating under an administrative system that, as FSANZ’s own impact analysis confirms (10), lacks the capacity to assess alternative forms of cultural evidence. Cultural authority in this context means that knowledge claims must be supported and verified by the Traditional Owners from whose Country and knowledge tradition the food originates, not simply asserted by any party wishing to commercialise a plant. I acknowledge that oral knowledges are not uniform across Indigenous groups and that questions of cultural authority in contested contexts require careful governance, which is precisely why Indigenous-led advisory structures are central to this approach rather than incidental to it. A detailed pathway for verifying this knowledge, including a tiered cultural authority framework, has been proposed elsewhere by the author of this paper (Figure 1) (1).

FSANZ has developed guidance material to help an enquirer prepare their submission for assessment, titled “Guidance Tool for Determining Whether a Food is Novel or Not” (19). The document asks four main questions to understand the history of use, including length of use, extent of use, quantity of use, and purpose or context of use. These guided questions are grounded in biocultural knowledge, yet the knowledge systems that are best positioned to answer them are not considered as primary source material. The current regulatory framework asks precisely the questions that Indigenous knowledge systems can answer, then refuses to accept it as evidence.

The argument presented here is not that empirical safety data is unnecessary. Instead, both biocultural evidence and empirical safety data should be applied where appropriate. Where cultural authority and an adequate understanding of a history of safe use can be established, further safety screening should be directed by the chemical composition of a food, particularly given that environmental conditions, including potential contaminant exposure from sources such as heavy metals, pesticides, or industrial pollutants, may impact dietary safety. This ensures the risk assessment remains proportionate to its actual risk profile rather than applied as a blanket standard. This is also where contemporary non-animal screening approaches are most appropriate. For many Indigenous peoples, animal testing to bring a food to market is often not culturally acceptable. It is also, in most cases, unnecessary: where a long record of human exposure already exists, that exposure is more direct evidence of safety in humans than an animal model can provide (33). The proportionality principle underpinning this approach has been explored and demonstrated in practice elsewhere (34).

Recognising traditional knowledge in the dietary safety assessment of traditionally used foods would not only address the immediate regulatory barrier but would represent a meaningful act of recognition for Indigenous food sovereignty.

## 4 Discussion

This Perspective extends nutritional ecology into the domain of food regulatory science. Nutritional ecology holds that diet, health, and safety cannot be understood in isolation from ecological and cultural context (2), yet this is precisely what current risk assessment does when it refuses to accept orally held knowledge as valid evidence of how a food has been used. A food assessed outside its biocultural context is not simply assessed in a culturally inappropriate way, it is assessed incompletely, because the very factors that determine its safety in practice, preparation technique, seasonal and environmental variation, combination with other foods, and the accumulated record of sustained human exposure, are the factors a decontextualised assessment discards. Recognising biocultural evidence is therefore not a concession to cultural sensitivity but a more scientifically complete application of nutritional ecology’s own foundational principles.

This reframing also resolves a practical failure of the current system. As argued above, self-assessment leaves Indigenous businesses carrying liability they are not equipped to manage, while a dedicated pathway grounded in biocultural evidence would serve consumers, producers, and FSANZ’s own public health mandate alike.

When Indigenous peoples can develop and work with their food systems on their own terms, opportunities to explore alternative sources of food and agricultural practice will follow. This also reframes the transition from traditional to commercial food environments not as a loss of food culture but as its continuation of culture on Indigenous terms. We have seen this pattern before. Aboriginal ranger programs across the continent demonstrate that when ecological knowledge is supported by economic incentive and purpose, knowledge systems flourish, culture is sustained, and measurable environmental outcomes follow (35, 36). Regulatory barriers that prevent Indigenous peoples from entering food markets on their own terms deny these outcomes and reform is one small but necessary step toward realising them.

The Aboriginal and Torres Strait Islander peoples of this continent hold the oldest living continuous cultural knowledge systems on earth, embedded across one of the world’s most biologically diverse landscapes (15, 37). Food regulatory agencies such as FSANZ have the opportunity to lead internationally by recognising rather than excluding these knowledge systems, developing a dedicated pathway for assessing traditionally used foods that centres Indigenous authority, recognises biocultural evidence, and aligns food regulation with whole-of-government commitments to support Indigenous self-determination. This paper does not prescribe a fully specified regulatory framework. That framework must be developed by FSANZ in genuine consultation with Indigenous peoples, Traditional Owners, and knowledge holders. What this paper provides are the principles that should govern that process and the evidence that such reform is both necessary and achievable.
